# Diagnostic delay in mental Experience of the psychiatric hospital Arrazi Salé Morocco

**DOI:** 10.1192/j.eurpsy.2023.1743

**Published:** 2023-07-19

**Authors:** S. Bahetta, N. Elmoussaoui

**Affiliations:** 1 Psychiatry, CHU RABAT SALE; 2 Psychiatry, Arrazi Psychiatric unversity hospital, Salé, Morocco

## Abstract

**Introduction:**

Mental illness is characterized by a major emotional, cognitive, and or behavioral impairment of an individual. It is usually accompanied by distress or functional impairments in important areas.

Mental illness affects 48.9% of the Moroccan population. This makes it a major public health issue but one that is still unrecognized and underestimated in the general population. Because of certain cultural aspects considering mental illness as a taboo or privileging traditional healing.

**Objectives:**

to evaluate the time between the initial symptomatology and the first psychiatric consultation Identify the course of action to be taken in the face of the first symptoms of the illness; Determine the factors responsible for the diagnostic delay;

**Methods:**

We conducted a cross-sectional study to assess the duration between initial symptoms and diagnosis and to identify the responsible factors of diagnostic delay. This study included 200 patients followed at the psychiatry department of the University Hospital Arrazi of Salé, and evaluated by an hetero questionnaire.

**Results:**

The average age of our patients was 29 years, male gender was predominant (84%). The mean diagnostic delay was 46 months. Data analysis showed some significant results: - The Diagnostic delay was longer in male patients. - The diagnsotic delay conditioned response to treatment and therefore the prognosis.

**Conclusions:**

In conclusion, public awareness of psychiatric problems, treatment availability, and educational efforts to overcome the social stigma are essential to reduce diagnostic delay and improve the prognosis of schizophrenia.

**Disclosure of Interest:**

None Declared

